# Supplementary material for: Antifungal Resistance and Genotyping of Clinical Candida parapsilosis Complex in Japan
Source: J Fungi (Basel). 2023 Dec 20;10(1):4. doi: 10.3390/jof10010004 (PMC10816931; doi:10.3390/jof10010004)
Supplement: Supplementary file 1 [file jof-10-00004-s001.zip › jof-2743072-supplementary.pdf]

Antifungal resistance and genotyping of clinical *Candid parapsilosis* complex in Japan

Hazim O. Khalifa[1,2,3#], Akira Watanabe[1], Katsuhiko Kamei[1]

[1] Division of Clinical Research, Medical Mycology Research Center, Chiba University, Chiba, Japan

[2]Department of Veterinary Medicine, College of Agriculture and Veterinary Medicine, United Arab Emirates University, Al Ain, P.O. Box 1555, United Arab Emirates

[3]Department of Pharmacology, Faculty of Veterinary Medicine, Kafrelsheikh University, Kafr El-Sheikh, Egypt

Running title: Acquired echinocandin resistance and genotyping of *C. parapsilosis*

[#]Address correspondence to Hazim O. Khalifa, hazimkhalifa@uaeu.ac.ae; hazem.khalifa1@vet.kfs.edu.eg

Supplementary Table S1. Characterization of clinical *C. parapsilosis* complex isolates recovered in this study*.

No.	IFM	Source	Age	Sex	Isolation date	Information	Notes	Prefecture
C. parapsilosis								
1	65757	Nail	73	F	April 2019	Nail candidiasis with black nails. Received RVCZ and fosravuconazole treatment.		Tokyo
2	65553	Catheter urine	0	F	October 2018	Very premature baby 25w 374g, from around 70d poor physical condition (fever, ascites), 80d intussusception laparotomy, 84d necrotizing enterocolitis resection, anasarca dehydration		Tokyo
3	65484	Blood	5	F	September 2018	Candidemia<br>Bulimia syndrome		Chiba
4	65480	Blood	Un	Un	2018	Candidemia		Chiba
5	65314	Cornea	Un	Un	2018	Infectious crystalline keratopathy. received quinolone eye drops and steroid eye drops for a long period of time.		Osaka
6	65134	Arterial blood, IVH tip	49	M	November 2017	Hemiplegia after cerebral hemorrhage.<br>Candidemia.	Same patient	Fukuoka
7	65133							
8	65119	Abscess	Un	Un	October 9 2017	Diabetes, necrosis of lower limbs.		Chiba
9	65072	Blood	68	M	May 14 2017	Inflammatory bowel disease (suspected ulcerative colitis).<br>Candidemia		Tochigi
10	64730	Blood, catheter backflow blood	55	M	September 2016	Distal myopathy.<br>Candidemia		Chiba

11	64459	Blood	92	F	September 2016	During IVH intubation. Candidemia		Gunma
12	63843	Blood	68	M	December 2015	Ileus dehydration. Candidemia History of MCFG use.		Chiba
13	63706	Blood	45	M	November 2015	Chronic active EB virus infection, NKT cell lymphoma. Candidemia. History of MCFG and AMPH + VRCZ treatment.		Chiba
14	63460	Blood	76	F	May 2015	Diabetes, pressure ulcers, pleural effusion. Candidemia.		Chiba
15	63795	Blood	76	M	March 2015	Candidemia. History of FLCZ treatment.		Chiba
16	63259	Liver	2	Un	January 2015	Congenital biliary atresia after liver transplantation. Suspected peritonitis.		Tokyo
17	63279	Blood	62	M	December 2014	Diabetes, infective endocarditis. Candidemia.		Osaka
18	62818	Catheter, venous blood	1	M	July 2014	Left chest wall vascular lymphangioma (Gorham-Stout syndrome). Candidemia.	Same patient	Kyoto
19	62817							
20	62626	Puncture fluid of renal pelvis	62	M	March 2014	Bilateral hydronephrosis, right ureteral stent placement. Suspected *Candida* urinary tract infection.		Chiba
21	62315	Blood	0	M	January 2014	Acute megakaryocyte leukemia. Candidemia. History of MCFG (preventive), Amphotericin (after fungal detection).		Akita

22	62624	Venous blood	64	M	October 2013	Gastric cancer.<br>Candidemia		Chiba
23	63792	Blood	77	M	September 2013	Myocardial infarction, cholangitis. Candidemia.		Chiba
24	63791	Blood	0	M	August 2013	Candidemia.<br>History of MCFG treatment		Chiba
25	62179	Venous blood	2	F	August 2013	Single ventricle, asplenia, fatal fasciitis.<br>Candidemia.		Chiba
26	62685	Arterial blood	40	M	May 2013	Severe acute pancreatitis.<br>Candidemia.		Chiba
27	62623	Venous blood	74	M	May 2013	Hypopharynx cancer, esophageal cancer.<br>Candidemia.		Chiba
28	62622	Venous blood	60	M	January 2013	Graves' disease, small intestinal stenosis.<br>Candidemia.		Chiba
29	62340	Blood	29	F	January 2013	Acute myeloid leukemia, catheter use, died in August 2013.<br>Candidemia.		Chiba
30	61882	Blood	53	F	January 2013	Died of a serious illness of Un cause. bacteria were also detected in aspirated sputum.<br>Candidemia		Chiba
31	61846	Eye (cornea)	78	M	January 2013	Corneal mycosis.<br>Rice harvesting foreign matter on the cornea.		Tokushima
32	61845	Eye (cornea)	68	M	January 2013	Corneal mycosis.<br>Foreign matter on the cornea.		Tokushima
33	61843	Eye (cornea)	61	M	January 2013	Corneal mycosis.<br>Foreign matter on the cornea during farm work.		Tokushima
34	61758	Venous blood	4	M	November 2012	Corneal mycosis.		Chiba

						Severe cerebral palsy, mechanical ventilation, tube feeding.		
35	61754	Blood	76	F	October 2012	Gastric ulcer, use of vascular catheter.		Chiba
36	63786	Blood	61	M	October 2012	Brain tumor.<br>History of Micafungin treatment.		Chiba
37	61750	Arterial blood	61	M	October 2012	Cholecystitis		Chiba
38	63785	Blood	0	M	July 2012	Candidemia.<br>History of Micafungin treatment.		Chiba
39	61434	Blood	80	M	July 2012	Diabetes.<br>Candidemia.		Tokyo
40	63784	Blood	78	M	January 2012	Diabetes, cerebral infarction.<br>Candidemia		Chiba
41	61746	Arterial blood	78	M	January 2012	Type 2 diabetes, transverse colon cancer, vascular catheter use, death		Chiba
42	61745	Venous blood	76	M	January 2012	Gastric cancer, Candidemia, vascular catheter use.		Chiba
43	63783	Blood	76	M	January 2012	Hypertension.<br>Candidemia.<br>History of Micafungin treatment.		Chiba
44	61192	Blood	73	M	December 2011	Suppurative spondylitis, uses IVH catheter.<br>Candidemia.		Chiba
45	61739	Venous blood	2	M	November 2011	CHARGE syndrome, use of vascular catheter, death on November 2011.		Chiba
46	61184	Vascular catheter	75	M	September 2011	Esophageal cancer.<br>Candidemia		Chiba
47	63782	Blood	72	M	September 2011	Maxillary cancer.<br>Candidemia		Chiba
48	63812	Blood	56	M	September 2011	Lung cancer.<br>Candidemia		Chiba

49	61194	Blood	11	F	September 2011	Traffic trauma (fracture, organ damage).<br>NB, intra-abdominal abscess Candida albicans was recovered.<br>Candidemia.<br>Antifungal drug use and oral anti-allergic drug for atopic dermatitis		Chiba
50	63781	Blood	65	M	August 2011	Candidemia		Chiba
51	61182	Venous blood	76	M	August 2011	Prostatic gland cancer, portal thrombosis, Disseminated intravascular coagulation (DIC). Candidemia.<br>Use of immunosuppressive medicine,		Chiba
52	61018	Blood	4	F	August 2011	Kawasaki disease, adenovirus infection.	Un	Chiba
53	61171	Vascular catheter	56	M	June 2011	Esophageal cancer, bronchial fistula. Candidemia	Un	Chiba
54	61008	Venous blood	59	M	March 2011	Ruptured aneurysm.	Un	Chiba
55	59620	Blood	84	M	July 2010	Ileus, intra-abdominal abscess. Candidemia.<br>Long-term use of vascular catheter.		Chiba
56	61201	Arterial blood	71	M	May 2010	Hepatocellular carcinoma.<br>Candidemia.<br>History of vascular catheter use.		Chiba
57	61199	Vascular catheter	51	M	April 2010	Cardia cancer.<br>History of vascular catheter use.		Chiba
58	61198	Vascular catheter	79	M	April 2010	Pneumococcal sepsis.<br>History of vascular catheter use.		Chiba
59	61002	Venous blood	76	F	March 2010	Cerebral infarction and tuberculosis,		Chiba
60	61001	Venous blood	56	M	February 2010	Diabetes and malignant lymphoma of the skin.		Chiba
61	61195	Vascular catheter	64	F	January 2010	Esophageal cancer.		Chiba
62	58051	Blood	65	F	July 2008	Unknown condition		Kyoto

						The patient received MCFG and VRCZ.		
63	55019	Blood	2	F	July 2006	Patient developed autoimmune enteritis at the age of one.<br>Disseminated intravascular coagulation (twice), multiple organ failure, and fungi suspected to be *Candida* were found in blood cultures.<br>The patient received prednisolone, azathioprine in the abdomen, in addition to FCZ, MCFG, and VCZ.		Aichi
64	54789	Un	Un	Un	July 2005	After the accident, skin grafting was performed in childhood, but a granulomatous rash appeared and did not heal.		Gifu
65	64439	Cornea	Un	Un	Un	Un		Un
C. metapsilosis								
66	66283	Blood	71	M	November 2019	T-cell lymphoma		Kanagawa
67	64455	Blood	0	F	November 2019	Acute lymphocytic leukemia		Chiba
68	63811	Blood	56	M	September 2011	Lung cancer		Chiba
69	63788	Blood	81	M	May 2013	Duodenal ulcer, cerebral infarction<br>History of MCFG treatment.		Chiba
70	63787	Blood	59	M	November 2012	No information<br>History of MCFG treatment.		Chiba
71	52586	otorrhea	Un	Un	Un	Un		Tokyo
72	52581	otorrhea	Un	Un	Un	Un		Tokyo
73	52523	otorrhea	Un	Un	Un	Un		Tokyo
74	52516	Pus	Un	Un	Un	Un		Tokyo
C. orthopsilosis								
75	65317	Blood	14	F	2018		Same patient	Saitama

76	65316					Neuroblastoma, acute myeloid leukemia and death		
77	65099	Venous blood	79	M	October 2017	Ischemic colitis after abdominal aortic aneurysm surgery. History of FLCZ treatment.		Chiba
78	52485	Feces	Un	Un	Un	Un	Un	Tokyo
79	52457	Pharyngeal secretion	Un	Un	Un	Un	Un	Tokyo

* Abbreviation: M, male; F, Female; Un, Known.

Supplementary Table S2. Oligonucleotides used in this study for PCR and microsatellite genotyping experiments.

Primer name	Sequence	Target gene/Purpose	PCR product sizes	Reference
CP_ERG_FW	CCCATCGTTTAAAACATCCAAAGACCTTAGC	Cp*ERG11*/PCR and sequencing	1921 bp	This study
CP_ERG_SQ1	GGAAAAGGTGTTATTTCGATTGTCCG	Cp*ERG11*/sequencing		This study
CP_ERG_SQ2	TTGATTGGTGTTTTGATGGGAGGACAGC	Cp*ERG11*/sequencing		This study
CP_ERG_RV	GAATTGTGGTGGTTTTACTTAGATAATTGGTG	Cp*ERG11*/PCR and sequencing		This study
CO_ERG_FW	GTAATCAAAGTGTTTCCTAT	Co*ERG11*/PCR and sequencing		35
CO_ERG_SQ1	GTGGTGTTGTTGATGTATTGCAATCGC	Co*ERG11*/sequencing		This study
CO_ERG_SQ2	GCCATTGGTAAACAACACCATCAAAG	Co*ERG11*/sequencing		This study
CO_ERG_RV	ACGGGGTTACACTGATTCTA	Co*ERG11*/PCR and sequencing		35
CP_HS1,2_FW	GCTGTCATGCCTTTGGGAGGTT	Cp*FKS1* (HS1 and HS2)/PCR and sequencing	2633 bp	This study
CP_HS1_RV	GTCAATAGCTAACAAATGTTCTCTGTACATGGA	Cp*FKS1* (HS1)/sequencing		This study
CP_HS2_FW	AGAGAATATTACTACTTGTCAACTCAATTGCC	Cp*FKS1* (HS2)/sequencing		This study
CP_HS1,2_RV	CTAGCACCCATATAAATTGACGAGTCAGC	Cp*FKS1* (HS1 and HS2)/PCR and sequencing		This study
CM_HS1,2_FW	GCCGGATTGACCTTGTATTCCAAGTCGG	Cm*FKS1* (HS1 and HS2)/PCR and sequencing	2611 bp	This study
CM_HS1_RV	GGTAATCTGGTGAAAATATTTCTCCATGGTGTC	Cm*FKS1* (HS1)/sequencing		This study
CM_HS2_FW	GGTGCCGGTATGGGTGAACAGATGTTGTC	Cm*FKS1* (HS2)/sequencing		This study
CM_HS1,2_RV	CAACAGTCATATCAGTAAAGACAGATGATGAATAG	Cm*FKS1* (HS1 and HS2)/PCR and sequencing		This study
CO_HS1,2_FW	CTTCGCTGTTATGCCTTTGGGAGGATTG	Co*FKS1* (HS1 and HS2)/PCR and sequencing	2610 bp	This study

CO_HS1_RV	GAGTAGATTCTCTTTGGCAATCTGGTG	Co*FKS1* (HS1)/sequencing		This study
CO_HS2_FW	GTCATTCTACTATGGTCACCCAGGTTTCC	Co*FKS1* (HS2)/sequencing		This study
CO_HS1,2_RV	GCGAAACGCGAATACAAGATGGAGAATGGG	Co*FKS1* (HS1 and HS2)/PCR and sequencing		This study
CP1-F	VIC 5´ AAAGTGCTACACACGCATCG	Microsatellite genotyping of *C. parapsilosis*		18
CP1-R	GGCTTGCAATTTCATTTCCT			
CP4-F	PET 5´ CAAATCATCCAGCTTCAAACC			
CP4-R	CATCAAACAAGAATTCGATATCAC			
CP6-F	FAM 5´ CAGGAACAGGACAATGGTGA			
CP6-R	TCTGGAGCCTCTAGGACGTTT-			
B5-F	FAM 5´ AGGTTTGTAGTAGTGTCCCTATGG			
B5-R	TATCTCTCTCGCCATTTGAACG			

Supplementary Table S3. MIC values of all tested isolates against wide range of antifungal agents.

No	IFM	MFG	CAS	AMB	5FC	FLC	ITC	VRC	MZ
C. parapsilosis									
1	65757	1	1	0.5	≤0.12	0.5	0.06	≤0.015	0.25
2	65553	0.5	0.5	0.5	≤0.12	0.25	0.03	≤0.015	0.06
3	65484	1	1	1	≤0.12	0.5	≤0.015	≤0.015	≤0.03
4	65480	1	1	1	≤0.12	0.5	0.03	≤0.015	0.06
5	65314	1	1	1	≤0.12	0.25	≤0.015	≤0.015	≤0.03
6	65134	0.5	0.25	1	≤0.12	0.5	0.03	≤0.015	0.06
7	65133	0.5	0.5	1	≤0.12	0.5	0.03	≤0.015	0.12
8	65119	0.5	0.5	1	≤0.12	0.5	0.06	≤0.015	0.06
9	65072	1	1	1	≤0.12	0.25	0.03	≤0.015	0.06
10	64730	1	1	1	≤0.12	0.5	0.06	≤0.015	0.06
11	64459	1	1	1	≤0.12	0.25	0.06	≤0.015	0.06
12	63843	1	1	1	≤0.12	0.25	0.03	≤0.015	0.06
13	63706	0.5	0.5	1	≤0.12	0.25	0.03	≤0.015	0.06
14	63460	1	1	0.5	≤0.12	0.25	0.03	≤0.015	0.06
15	63795	1	1	1	≤0.12	0.5	0.03	≤0.015	0.12
16	63259	0.25	0.5	1	≤0.12	0.25	≤0.015	≤0.015	≤0.03
17	63279	0.5	0.5	0.5	≤0.12	0.25	0.03	≤0.015	≤0.03
18	62818	1	1	1	≤0.12	0.25	0.03	≤0.015	≤0.03
19	62817	1	1	1	≤0.12	0.5	0.03	≤0.015	0.06
20	62626	1	1	1	≤0.12	0.25	≤0.015	≤0.015	≤0.03
21	62315	0.5	0.5	1	≤0.12	0.25	0.03	≤0.015	≤0.03
22	62624	0.5	0.5	1	≤0.12	0.5	≤0.015	≤0.015	≤0.03
23	63792	0.5	1	1	≤0.12	0.5	0.06	≤0.015	0.06
24	63791	1	1	1	≤0.12	0.25	0.03	≤0.015	0.06
25	62179	1	1	1	≤0.12	0.5	0.03	≤0.015	0.06
26	62685	1	1	1	≤0.12	1	0.12	0.06	0.25
27	62623	1	1	1	≤0.12	0.5	0.03	≤0.015	≤0.03
28	62622	0.5	1	1	≤0.12	0.5	0.06	0.03	0.06
29	62340	1	1	1	≤0.12	0.5	0.03	≤0.015	0.06
30	61882	1	1	1	≤0.12	0.25	0.03	≤0.015	≤0.03
31	61846	1	1	1	≤0.12	0.5	0.03	≤0.015	0.06
32	61845	1	1	1	≤0.12	0.5	0.06	0.03	0.12
33	61843	1	1	1	≤0.12	0.5	0.06	≤0.015	0.12
34	61758	1	1	1	≤0.12	0.5	0.03	≤0.015	0.06
35	61754	0.5	1	1	≤0.12	0.5	0.06	≤0.015	0.12
36	63786	1	1	1	≤0.12	0.5	0.06	≤0.015	0.12
37	61750	1	1	1	≤0.12	0.25	0.03	≤0.015	0.12
38	63785	1	1	1	≤0.12	0.5	0.03	≤0.015	0.06
39	61434	1	1	1	≤0.12	0.5	0.06	≤0.015	0.06

40	63784	1	1	1	≤0.12	0.5	0.03	≤0.015	0.06
41	61746	1	1	1	≤0.12	0.5	0.06	≤0.015	0.12
42	61745	1	1	1	≤0.12	0.5	0.03	0.03	0.12
43	63783	1	1	1	≤0.12	0.5	0.03	≤0.015	0.25
44	61192	0.5	1	1	≤0.12	0.12	0.06	≤0.015	0.25
45	61739	1	1	1	≤0.12	0.5	0.06	≤0.015	0.12
46	61184	1	1	1	≤0.12	0.5	0.06	0.03	0.12
47	63782	1	1	1	≤0.12	0.25	0.03	≤0.015	0.03
48	63812	1	1	1	≤0.12	0.25	0.03	≤0.015	0.06
49	61194	1	1	1	≤0.12	0.25	0.03	≤0.015	0.12
50	63781	1	1	0.5	≤0.12	0.25	0.06	≤0.015	0.12
51	61182	1	1	1	≤0.12	0.5	0.03	≤0.015	0.12
52	61018	1	1	1	≤0.12	0.25	0.03	≤0.015	0.06
53	61171	1	1	1	≤0.12	0.5	0.06	≤0.015	0.25
54	61008	1	1	1	≤0.12	0.5	0.06	0.03	0.12
55	59620	1	1	1	≤0.12	0.5	0.03	≤0.015	≤0.03
56	61201	1	1	1	≤0.12	0.5	0.03	≤0.015	0.06
57	61199	1	1	1	≤0.12	0.25	0.03	≤0.015	0.06
58	61198	1	1	1	≤0.12	0.5	0.03	≤0.015	≤0.03
59	61002	1	1	0.5	≤0.12	0.5	0.03	≤0.015	≤0.03
60	61001	1	1	1	≤0.12	0.5	0.06	0.03	0.06
61	61195	1	1	1	≤0.12	0.25	0.03	≤0.015	≤0.03
62	58051	0.5	1	1	≤0.12	0.25	0.03	≤0.015	≤0.03
63	55019	1	1	1	≤0.12	1	0.12	0.03	0.25
64	54789	1	1	0.5	≤0.12	0.25	0.03	≤0.015	0.06
65	64439	0.25	0.5	1	≤0.12	2	0.06	0.03	0.12
C. metapsilosis									
66	66283	0.06	0.5	0.5	≤0.12	1	0.06	0.03	0.25
67	64455	0.25	0.5	1	≤0.12	0.5	0.12	0.03	0.12
68	63811	0.25	0.5	1	≤0.12	2	0.12	0.03	0.25
69	63788	0.25	0.5	1	≤0.12	2	0.12	0.03	0.25
70	63787	0.25	0.5	1	≤0.12	2	0.12	0.03	0.25
71	52586	0.25	0.5	1	≤0.12	4	0.06	0.06	0.25
72	52581	0.25	0.5	1	≤0.12	1	0.12	0.03	0.25
73	52523	0.25	0.5	1	≤0.12	1	0.12	0.03	0.12
74	52516	0.25	0.5	1	≤0.12	1	0.06	0.03	0.12
C. orthopsilosis									
75	65317	0.25	0.5	1	≤0.12	0.5	0.06	≤0.015	0.12
76	65316	0.5	0.5	0.5	≤0.12	0.5	0.06	≤0.015	0.12
77	65099	0.25	0.5	1	≤0.12	0.5	0.12	0.03	0.25
78	52485	0.12	0.5	0.5	≤0.12	0.5	0.06	≤0.015	0.12
79	52457	0.25	0.5	1	≤0.12	2	0.12	0.03	0.25

Supplementary Table S4. Mutations detected in *ERG11* gene for both *C. parapsilosis* and *C. orthopsilosis*.

No	IFM	*ERG11 synonymous mutations*	*ERG11 nonsynonymous mutations*
C. parapsilosis			
1	65757	T591C	
2	65553	T591C	
3	65484	T591C	
4	65480	T591C	R398I
5	65314	T591C	
6	65134	T591C	
7	65133	T591C	
8	65119	T591C	R398I
9	65072	T591C	R398I
10	64730	T591C	
11	64459	T591C	R398I
12	63843	T591C	
13	63706	T591C	
14	63460	T591C	R398I
15	63795	T591C	R398I
16	63259	T591C	
17	63279	T591C	
18	62818	T591C	
19	62817	T591C	
20	62626	T591C	
21	62315	T591C	
22	62624	T591C	
23	63792	T591C	R398I
24	63791	T591C	
25	62179	T591C	
26	62685	T591C	
27	62623	T591C	R398I
28	62622	T591C	R398I
29	62340	T591C	
30	61882	T591C	R398I
31	61846	T591C	R398I
32	61845	T591C	R398I
33	61843	T591C	R398I
34	61758	T591C	R398I
35	61754	T591C	
36	63786	T591C	R398I
37	61750	T591C	R398I

38	63785	T591C	R398I
39	61434	T591C	
40	63784	T591C	R398I
41	61746	T591C	R398I
42	61745	T591C	R398I
43	63783	T591C	R398I
44	61192	T591C	R398I
45	61739	T591C	R398I
46	61184	T591C	R398I
47	63782	T591C	
48	63812	T591C	
49	61194	T591C	R398I
50	63781	T591C	
51	61182	T591C	
52	61018	T591C	R398I
53	61171	T591C	
54	61008	T591C	R398I
55	59620	T591C	
56	61201	T591C	R398I
57	61199	T591C	R398I
58	61198	T591C	
59	61002	T591C	R398I
60	61001	T591C	R398I
61	61195	T591C	R398I
62	58051	T591C	R398I
63	55019	T591C	
64	54789	T591C	
65	64439	T591C	
		C. orthopsilosis	
66	65317		Y13C, F420S
67	65316		Y13C, F420S
68	65099		Q211K, F420S, A421V, V481I
69	52485		Y13C, F420S
70	52457		Y13C, F420S

Supplementary Table S5. *Candida parapsilosis* strain typing results using microsatellite genotyping.

Genotype	No. of isolates (patients) per genotype	Concatenated diploid genotype
1.	4 (4)	252252376442136136218313
2.	3 (3)	247255313313140140273273
3.	2 (2)	245250313313134140269287
4.	1 (1)	229294313313136136273292
5.	1 (1)	229294313313156156273292
6.	1 (1)	302247346346136136218218
7.	1 (1)	252247329326137139257271
8.	1 (1)	245250313313140140287287
9.	1 (1)	250252376376136136307307
10.	1 (1)	230250289289136162275295
11.	1 (1)	248250376445136136218340
12.	1 (1)	230252376376136136310310
13.	1 (1)	216248320320140140287287
14.	1 (1)	245250313313140140269287
15.	1 (1)	230250376376134134275325
16.	1 (1)	250250376439136136218218
17.	1 (1)	245250313313142142255255
18.	1 (1)	245250313313140140269269
19.	1 (1)	248250253313156156278278
20.	1 (1)	248248313313152152264264
21.	1 (1)	230250376376136136275325
22.	1 (1)	245252313313140140272272
23.	1 (1)	228228264336118118298298
24.	1 (1)	248250358358136136278298
25.	1 (1)	250250379479136136218310
26.	1 (1)	250250397397136136272272
27.	1 (1)	248252313313140140271287
28.	1 (1)	248250403403134146293307
29.	1 (1)	250259376423136136218310
30.	1 (1)	230250382382136136310310
31.	1 (1)	250250376376136136218218
32.	1 (1)	250250379379136136218218
33.	1 (1)	248248317317116116298298
34.	1 (1)	252252400400136136310310
35.	1 (1)	250250345345116116296296
36.	1 (1)	252252376442136136310310
37.	1 (1)	232252376376136136340340
38.	1 (1)	252252376391146146307307
39.	1 (1)	232252376407136162276296
40.	1 (1)	250250332332154154265265
41.	1 (1)	232252379379136136279279
42.	1 (1)	252252291326152152298298
43.	1 (1)	252252373442136136218218
44.	1 (1)	241269313313197197296296

45.	1 (1)	252252346386138138218276
46.	1 (1)	252252376376136136293298
47.	1 (1)	247252313313140140271287
48.	1 (1)	255255376376136136293307
49.	1 (1)	255255379379136136213293
50.	1 (1)	232252376376136136276328
51.	1 (1)	250250400400134146290325
52.	1 (1)	252252316316136136298298
53.	1 (1)	232252376376136162276296

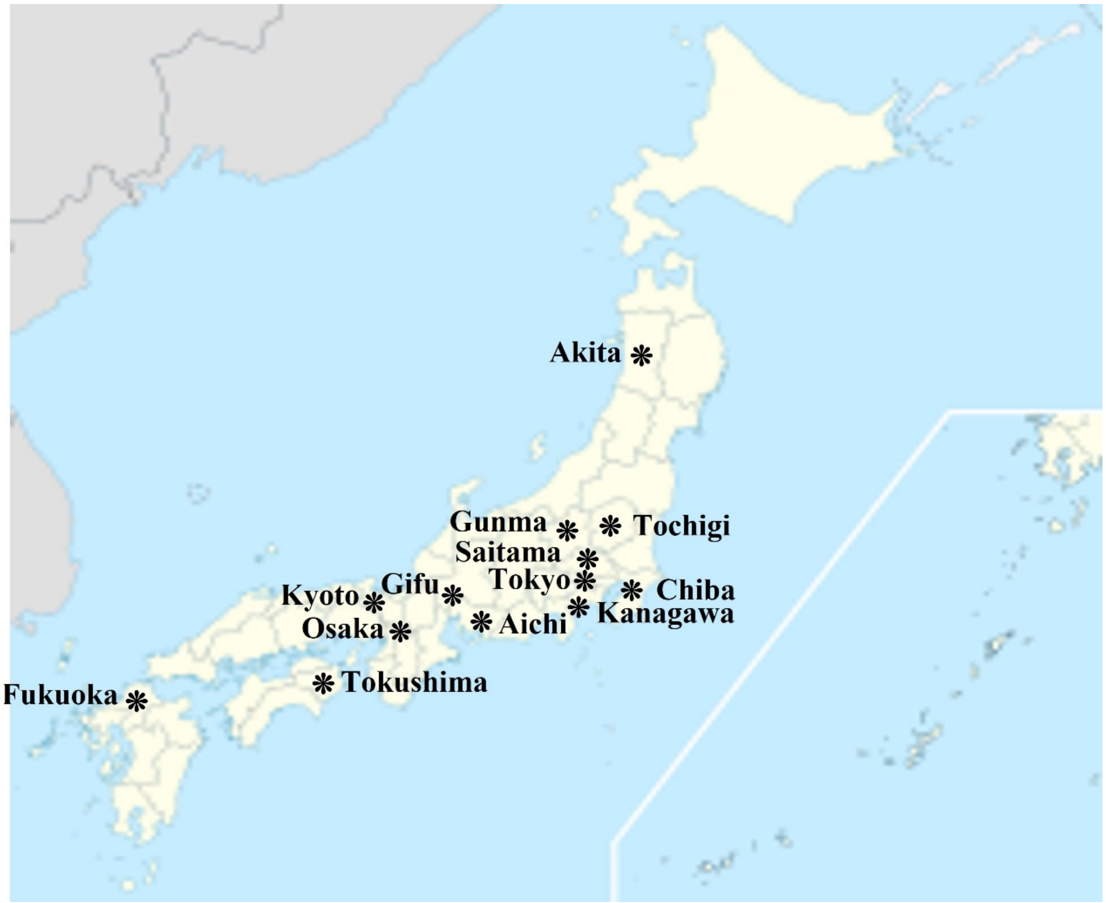

Supplementary Figure S1. Map of the location of isolate collections.

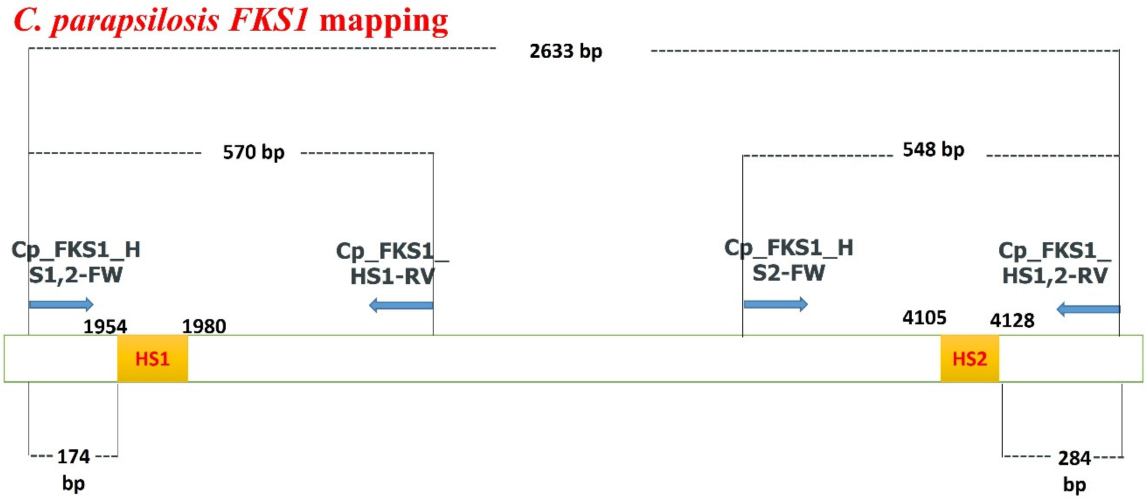

Supplementary Figure S2. Primer mapping for PCR and sequencing of *C. parapsilosis FKS1* hot spot regions.

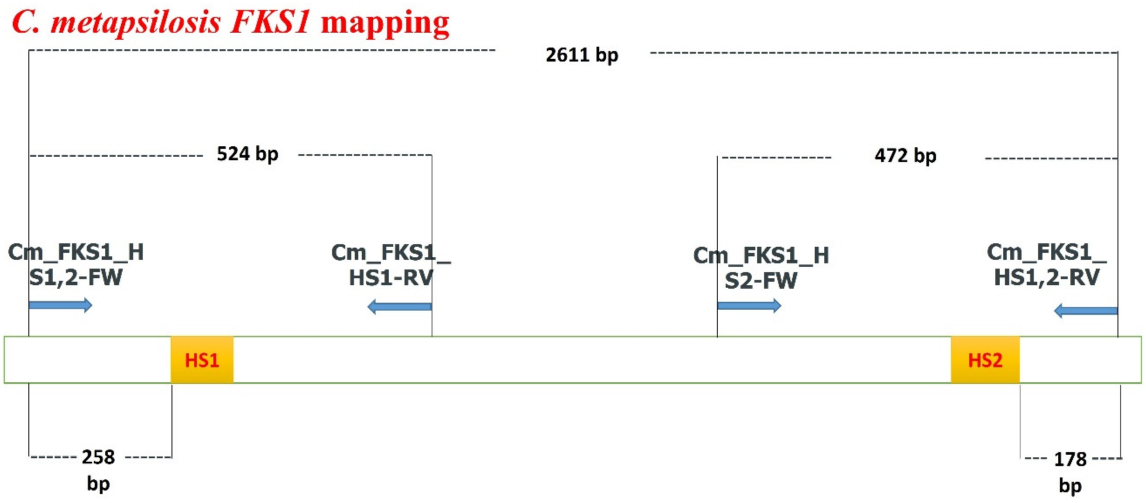

Supplementary Figure S3. Primer mapping for PCR and sequencing of *C. metapsilosis FKS1* hot spot regions.

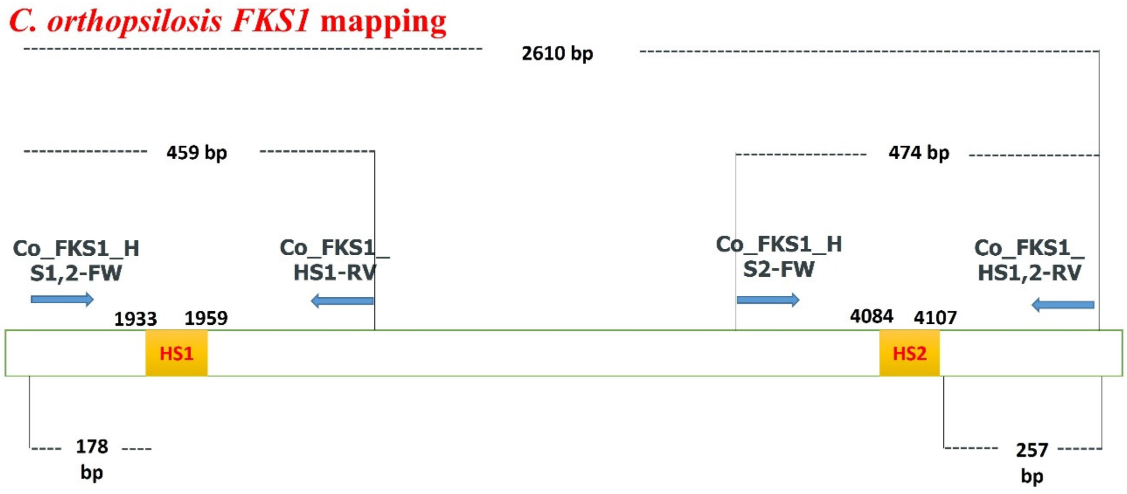

Supplementary Figure S4. Primer mapping for PCR and sequencing of *C. orthopsilosis FKS1* hot spot regions.

References:

18- Sabino, R.; Sampaio, P.; Rosado, L.; Stevens, D.A.; Clemons, K.V.; Pais, C. New microsatellite markers able to distinguish among *Candida parapsilosis Sensu Stricto* isolates. J. Clin. Microbiol. 2010, 48, 1677–1682. https://doi.org/10.1128/JCM.02151-09.

35- Rizzato C, Poma N, Zoppo M, Posteraro B, Mello E, Bottai D, Lupetti A, Sanguinetti M, Tavanti A. CoERG11 A395T mutation confers azole resistance in *Candida orthopsilosis* clinical isolates. J. Antimicrob. Chemother. 2018, 73, 1815–1822.
